# Identification of the Optimal Light Harvesting Antenna Size for High-Light Stress Mitigation in Plants

**DOI:** 10.3389/fpls.2020.00505

**Published:** 2020-05-15

**Authors:** Guangxi Wu, Lin Ma, Richard T. Sayre, Choon-Hwan Lee

**Affiliations:** ^1^Department of Molecular Biology, Pusan National University, Busan, South Korea; ^2^Pebble Labs, Los Alamos, NM, United States; ^3^New Mexico Consortium, Los Alamos, NM, United States

**Keywords:** antenna size, biomass yield, chlorophyll *b*, photosynthesis, reactive oxygen species, stress

## Abstract

One of the major constraints limiting biomass production in autotrophs is the low thermodynamic efficiency of photosynthesis, ranging from 1 to 4%. Given the absorption spectrum of photosynthetic pigments and the spectral distribution of sunlight, photosynthetic efficiencies as high as 11% are possible. It is well-recognized that the greatest thermodynamic inefficiencies in photosynthesis are associated with light absorption and conversion of excited states into chemical energy. This is due to the fact that photosynthesis light saturates at one quarter full sunlight intensity in plants resulting in the dissipation of excess energy as heat, fluorescence and through the production of damaging reactive oxygen species. Recently, it has been demonstrated that it is possible to adjust the size of the light harvesting antenna over a broad range of optical cross sections through targeted reductions in chlorophyll *b* content, selectively resulting in reductions of the peripheral light harvesting antenna size, especially in the content of Lhcb3 and Lhcb6. We have examined the impact of alterations in light harvesting antenna size on the amplitude of photoprotective activity and the evolutionary fitness or seed production in Camelina grown at super-saturating and sub-saturating light intensities to gain an understanding of the driving forces that lead to the selection for light harvesting antenna sizes best fit for a range of light intensities. We demonstrate that plants having light harvesting antenna sizes engineered for the greatest photosynthetic efficiency also have the greatest capacity to mitigate high light stress through non-photochemical quenching and reduction of reactive oxygen associated damage. Under sub-saturating growth light intensities, we demonstrate that the optimal light harvesting antenna size for photosynthesis and seed production is larger than that for plants grown at super-saturating light intensities and is more similar to the antenna size of wild-type plants. These results suggest that the light harvesting antenna size of plants is designed to maximize fitness under low light conditions such as occurs in shaded environments and in light competition with other plants.

## Introduction

In nature, photosynthetic organisms grow under constantly varying light intensities ranging from full sunlight intensities to sub-saturating light intensities. In addition, leaves at the top of the canopy experience higher light intensities than those at the bottom of the canopy. This raises the question why do virtually all plants have light harvesting antenna sizes that capture photons at rates nearly 10-fold greater than they can be converted into chemical energy at full sunlight conditions. Having large light harvesting antenna sizes incurs damage to the photosynthetic apparatus at light intensities greater than those that saturate electron transfer processes. Plants mitigate high light (HL) stress through non-productive energy dissipation pathways including heat and fluorescence and the production of damaging reactive oxygen species (Perrine et al., 2012; Friedland et al., 2019). This raises the question why have plants evolved large fixed size light harvesting antenna sizes that light saturate at one quarter full sunlight intensity.

In low light environments and in stratified plant canopies having a large wasteful light harvesting antenna may provide a selective advantage by excluding light from competing species. In monoculture or agricultural environments, however, having large wasteful light harvesting complexes (LHCs) may not be advantageous for biomass production (Ort et al., 2011). For example, it has been shown in green algal and plant monocultures that organisms which have slightly reduced light harvesting apparatus grow more efficiently than wild-type (WT) strains having larger, more wasteful light harvesting antenna (Perrine et al., 2012; Friedland et al., 2019). Chlamydomonas lines having smaller optimized light harvesting antenna sizes were shown to have growth rates 40% greater than WT algae when grown in simulated pond environments (Perrine et al., 2012). Similar effects of antenna size reduction on enhanced crop biomass production have been observed in Camelina engineered to have optimal light harvesting antenna sizes (Friedland et al., 2019).

Chlorophyll (Chl) *b* accounts for approximately half of the chlorophyll in the peripheral LHC and is not present in photosynthetic reaction centers. The LHC apoproteins which bind Chl *b* and other pigments are made in the cytoplasm, imported into chloroplasts, and folded in the presence of the photosynthetic pigments. As a result, a reduction or absence of Chl *b* can reduce the stability of the LHC proteins resulting in their degradation and graded reductions in the apparent optical cross section of the light harvesting antenna (Hoober et al., 2007; Friedland et al., 2019). As previously demonstrated, small reductions in Chl *b* synthesis (Chl *a/b* ratio = 5) leads to a reduction in the number of trimeric LHCII complexes. Reductions in Chl *b* levels leading to Chl *a/b* ratios > 6.5, however, result in additional losses in photochemical efficiency and the ability to dissipate excess excited states at saturating light intensities (Perrine et al., 2012; Friedland et al., 2019). Thus, there is an optimal light harvesting antenna size for plants corresponding to a Chl *a/b* ratio of 5.

The fact that smaller light harvesting antenna are more susceptible to photodamage than larger antenna is counter-intuitive since reductions in light harvesting antenna size would inherently be expected to reduce HL stress damage as a result of the decrease in light capture efficiency. Thus, it is hypothesized that there is likely a trade-off between reductions in photosynthetic efficiency and reductions in HL stress induced damage associated with alterations in light harvesting antenna size. To determine the optimal light harvesting antenna size for biomass production and fitness (seed production) under low and high light conditions, we characterized the photosynthetic performance and light stress responses of Camelina plants having altered levels of Chl *b* accumulation and associated light harvesting antenna sizes. These plants had Chl *a/b* ratios ranging from 3 to 14 and corresponding alterations in light harvesting antenna size (Friedland et al., 2019).

We demonstrate that for plants having an optimal antenna size for photosynthetic efficiency, the photo-protective mechanisms are fully operational resulting in the best overall photosynthetic performance. In contrast, plants having reduced light harvesting antenna sizes (Chl *a/b* ratios > 6.5) are more susceptible to HL damage. Thus, there is a tipping point in light harvesting antenna size at which reductions in light harvesting antenna size leads to both reductions in photosynthetic efficiency and reductions in photoprotective mechanisms against HL leading to reductions in both electron transport and high light stress protection efficiency (Friedland et al., 2019). In contrast, the optimal light harvesting antenna size for photosynthesis and seed production for plants grown at low light intensities is much larger and more similar in size to the light harvesting antenna of WT plants. These results suggest that for Camelina light harvesting antenna sizes in wild-type plants have been selected for best performance under low light intensities as occurs during competition for light.

## Materials and Methods

### Plants and Growth Condition

Wild-type *Camelina sativa* plants and T4 generation back-crossed transgenic plants expressing RNAi molecules targeting the silencing of the chlorophyllide *a* oxygenase (CAO) gene previously described by Friedland et al. (2019) were grown in the greenhouse at 24°C/26°C with a 14 h/10 h day/night photoperiod. The average moderate light intensity (ML) at mid-morning in the green house was 850 μmol photons m^–2^ s^–1^ (400–700 nm, photosynthetic active radiation, PAR), while for shaded low light (LL) plants the growth light intensity was sub-saturating (200 μmol photons m^–2^ s^–1^, PAR). Fully expanded leaves from the top of WT and CAO RNAi (CR) plants were assessed using 3- to 5-weeks old plants for all experiments. The Chl concentration was determined in aqueous 80% acetone as described by Porra et al. (1989). The transgenic plants were assigned to three different groups according to their Chl *a/b* ratios or apparent light harvesting antenna sizes at 3–5 weeks of age including: a low-intermediate Chl *a/b* ratio group (CR L-I) having Chl *a/b* ratios ranging from 4.5 to 6.5, a high-intermediate Chl *a/b* ratio group (CR H-I) group having Chl *a/b* ratios ranging from 6.5 to 8.5, and a very-high Chl *a/b* ratio group (CR V-H) having Chl *a/b* ratios greater than 8.5 having the smallest light harvesting antenna size. Significantly, the Chl *a/b* ratios of top fully expanded leaves from a given plant line did not significantly change during growth from 3 to 5 weeks, indicating antenna sizes were stable in a given transgenic line (Supplementary Figure S1).

### Light Stress Treatment

Leaves were detached from dark-adapted (overnight) plants and floated on water to avoid water stress for all subsequent treatments. Leaves were then exposed to different light intensities ranging from 200, 600, 1,000, 1,400 and 1,800 μmol photons m^–2^ s^–1^ PAR using a white light-emitting diode (LED) lamp for 3 h at 26°C. For HL treatments, plants were dark-adapted overnight and treated at 1,000 μmol photons m^–2^ s^–1^ PAR using the LED lamp for 24 h at 26°C.

### Chlorophyll Fluorescence Measurements

*In vivo* chlorophyll fluorescence kinetics were measured using detached leaves using a Handy FluorCam FC 1000-H (Photon Systems Instruments, Drásov, Czechia) after dark-adaptation for 30 min at room temperature. The photosystem II (PSII) photochemical efficiency (Fv/Fm) was calculated according to the equation of Fv/Fm = (Fm – Fo)/Fm, where Fo is the minimum fluorescence determined using low-intensity measuring light pulses at 620 nm, and Fm is the maximum fluorescence determined after a 0.8 s saturating pulse of white light at 4,000 μmol photons m^–2^ s^–1^. Non-photochemical quenching (NPQ) was calculated according to the equation of NPQ = (Fm – Fm′)/Fm′, where Fm’ is the maximum fluorescence measured using a light-adapted leaf. For the measurement of NPQ development kinetics, leaves were exposed to actinic light (100 μmol photons m^–2^ s^–1^) for 60 s and were then kept in darkness for 60 s before measurement of NPQ decay kinetics. To determine the impact of high light stress on NPQ kinetics and its long-term recovery, plants were exposed to high light (1,000 μmol photons m^–2^ s^–1^) for 3, 6, 9 and 24 h. After dark-adaptation for 30 min NPQ kinetics were determined as described above.

### Reactive Oxygen Species (ROS) Measurements

A qualitative or histochemical assay for superoxide oxygen detection was performed using detached leaf segments as previously described by Fryer et al. (2002) and Zulfugarov et al. (2011). Briefly, leaf samples from overnight dark-adapted plants were immersed in 6 mM nitroblue tetrazolium (NBT) solution containing 50 mM HEPES buffer (pH 7.5) for 2 h in the dark. After the treatment at the corresponding growth light conditions (ML and LL) for 4 h, pigments were extracted from leaf segments using absolute ethanol at 65°C by shaking in a water bath. Quantitative levels of NBT reactive superoxide radicals produced were then analyzed from the image in the gray scale value using Image J^1^.

### Measurement of Light Stress Induced Lipid Peroxidation

Malondialdehyde (MDA) is a product of lipid peroxidation and an indirect indicator of ROS-mediated membrane lipid damage. MDA production was determined using the thiobarbituric acid reaction according to Peever and Higgins (1989) with slight modifications. Leaves were detached from overnight dark-adapted plants, floated on water, and dark-adapted as controls or illuminated at the corresponding growth light intensity for 4 h. Leaf material (100 mg) was then homogenized in 1 mL 0.1% (w/v) trichloroacetic acid in a blender at 4°C. The homogenate was centrifuged at 10,000 *g* for 10 min. After adding 1 mL of 20% trichloroacetic acid containing 0.6% (w/v) thiobarbituric acid to the supernatant, the mixture was incubated at 95°C for 25 min and then centrifuged at 10,000 *g* for 10 min. The absorbance was measured from the supernatant at 440, 532, and 600 nm. MDA contents were calculated as described by Hodges et al. (1999).

### Sodium Dodecyl Sulfate-Polyacrylamide Gel Electrophoreses and Immunoblotting

Sodium dodecyl sulfate (SDS)-polyacrylamide gel electrophoreses (PAGE) and immunoblotting were performed as described by Towbin et al. (1979). Thylakoid membrane containing 2 μg Chl was solubilized with SDS sample buffer containing 40 mM Tris-HCl (pH 6.8), 10% (v/v) glycerol, 0.1% (w/v) bromophenol blue, 0.1M dithiothreitol and 2% (w/v) SDS for 30 min at room temperature. Polypeptides were separated by SDS-PAGE using 12% (w/v) acrylamide gel with 3M urea and electro-transferred into polyvinylidene fluoride membrane (Immobilon-P, Merck, Darmstadt, Germany). Photosystem I (PSI), PSII and LHC proteins were detected using antibodies raised against the PsbA or D1 protein, PsbS, PsaA, Lhcb1-Lhcb6, and Lhca1-Lhca4 (Agrisera AB, Vännäs, Sweden). After incubating with anti-rabbit IgG HRP conjugated secondary antibody (Agrisera, Vännäs, Sweden) for 2 h at room temperature, antibody-specific signals were imaged using a Clarity Western ECL substrate (Bio-Rad Laboratories, Berkeley, CA, United States).

### Pigments Analysis

Xanthophyll cycle pigment were determined according to Gilmore and Yamamoto (1991) with slight modifications. Pigments were extracted with 100% cold acetone from overnight dark-adapted leaves after treatment with HL (1,000 μmol photons m^–2^ s^–1^) for 0, 1, and 2 h. The pigment extracts were filtered through a 0.2-μm membrane filter. Pigment separation was performed in a high performance liquid chromatography system (HP 1100 series, Hewlett-Packard, Waldbronn, Germany) on a Spherisorb ODS-1 column (Waters, Milford, MA, United States) using a solvent mixture of acetonitrile:methanol:0.1M Tris-HCl pH 8.0 (72:12:7, v/v/v) for 6 min followed by a 10 min linear gradient to methanol:hexane (4:1, v/v). The eluted pigments were monitored at 445 nm. Concentrations of the pigments were estimated by using the conversion factors for peak area normalized to 100% Chl *a* molecule (Chen et al., 2012).

### Statistical Analysis

All experiments were repeated at least three times (*n* ≥ 3). Values are expressed as mean ± SD. The significance of differences between experimental groups was analyzed using the unequal variance two-tailed Student’s *t*-tests. Statistically significant are considered as ^∗^*P* < 0.05 or ^∗∗^*P* < 0.01.

## Results

### Sensitivity of NPQ and PSII to HL Stress Damage as a Function of Light Harvesting Antenna Size

**FIGURE 1:**
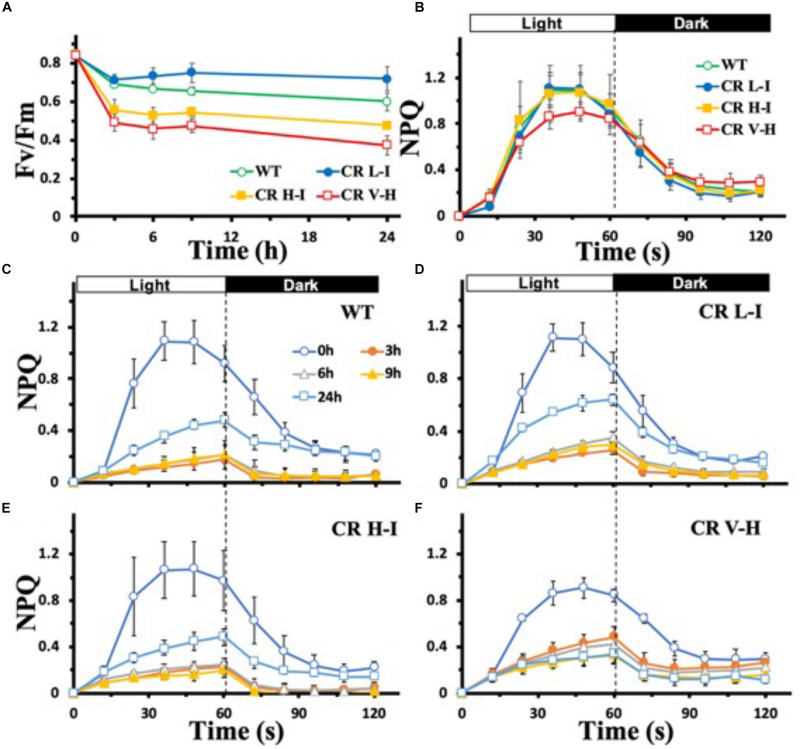
Comparison of Fv/Fm and NPQ development and relaxation kinetics in wild-type and CAO RNAi transgenic lines under high light stress. Wild-type (WT), CR L-I (Chl *a/b* = 4.5–6.5), CR H-I (Chl *a/b* = 6.5–8.5), and CR V-H (Chl *a/b* 8.5 or above) plant leaves were dark-adapted for 30 min before the measurement of Fv/Fm **(A)** and NPQ **(B–F)**. Leaves were exposed to high light (HL) stress at 1,000 μmol photons m^–2^ s^–1^ for 24 h. For the measurement of NPQ development and relaxation kinetics, leaves were exposed to actinic light (100 mmol photons m^–2^ s^–1^) for 60 s and were then kept in darkness for 60 s. The effects of HL stress on the NPQ development and relaxation kinetics were tested for 24 h in WT **(C)**, CR L-I **(D)**, CR H-I **(E)**, and CR V-H **(F)** lines. All experiments were done using fully expanded leaves from the top of 4–5 weeks old plants. Results represent the average and SD of three independent measurements.

To determine the sensitivity of plants having different light harvesting antenna sizes to HL stress, we analyzed the impact of HL stress (1,000 μmol photons m^–2^ s^–1^ for 24 h) on PSII photochemical efficiency as determined by the Chl fluorescence Fv/Fm ratio (Figure 1A). In contrast to plants with substantially reduced light harvesting antenna sizes (Chl *a/b* > 6.5), plants with optimal light harvesting antenna sizes (CR L-I) had lower reductions in photochemical efficiency following light stress treatment. In fact, the lowest reductions in photochemical efficiency following HL stress treatment were observed in CR L-1 plants and not in wild-type plants with larger light harvesting antenna complexes.

To gain greater insights into the biophysical basis for these differences in light stress sensitivity associated with different light harvesting antenna sizes, we compared the relative levels of dark-adapted non-photochemical quenching (NPQ) activity of plants having a range of light harvesting antenna sizes (Figure 1B). NPQ is one of the mechanisms by which excess Chl excited states are dissipated non-destructively (Zulfugarov et al., 2014). In overnight dark-adapted leaves, NPQ rise kinetics in all of the CAO RNAi lines were similar to that of WT, except for the CR V-H lines having the smallest light harvesting antenna size (Figure 1B). In addition, the maximum level of NPQ was reduced by 20% in the CR V-H lines and NPQ decay kinetics were slower than WT, CR L-I and CR H-I lines. Following high light exposure ranging from 3 to 9 h, NPQ development was repressed in all plants (Figures 1C–F). Recovery of NPQ after high light stress for 24 h also varied as a function of antenna size. In WT plants, the NPQ level reached after actinic light exposure for 60 s was 44% of the control level (Figure 1C). In CR L-I lines the NPQ level reached was 58% of the control level (Figure 1D), whereas the maximum NPQ level reached in CR H-I lines was only 45% of the control level (Figure 1E). The NPQ level reached in CR L-I lines was significantly higher than the levels reached both in WT and in CR H-I lines at *P* < 0.5. We observed that plants having the smallest antenna sizes (CR V-H lines) had no NPQ recovery after HL treatment for 24 h (Figure 1F). Thus, there was a tipping point in antenna size relative to NPQ recovery after HL stress with maximum recovery occurring in plants with Chl *a/b* ratios near 5, the Chl *a/b* ratio that is also optimal for photosynthetic efficiency (Friedland et al., 2019).

**FIGURE 2:**
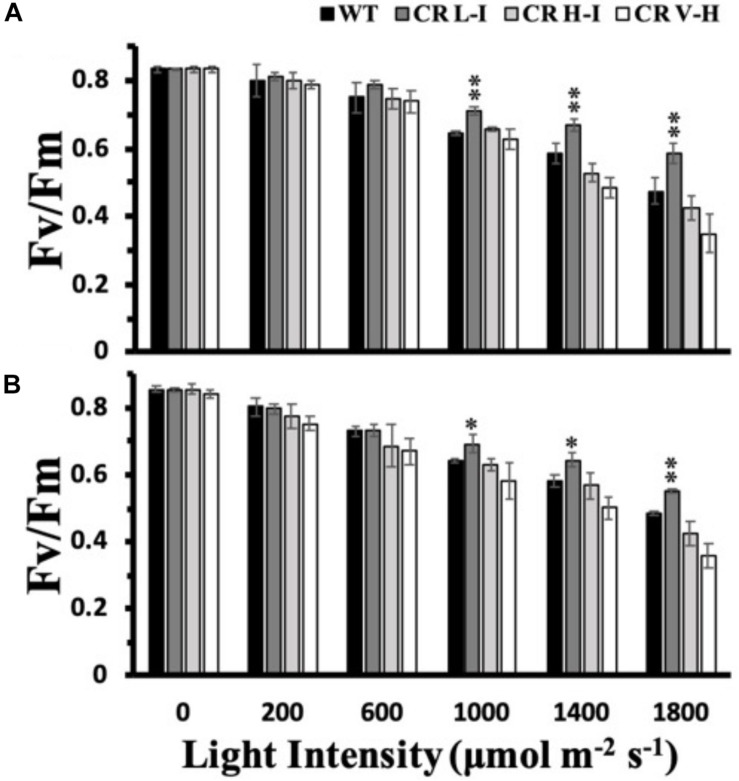
Comparison of growth light intensity-dependent changes in Fv/Fm in wild-type and CAO RNAi transgenic lines. Wild-type (WT), CR L-I (Chl *a/b* = 4.5–6.5), CR H-I (Chl *a/b* = 6.5–8.5), and CR V-H (Chl *a/b* 8.5 or above) plants grown either in moderate light (ML) (850 μmol photons m^–2^ s^–1^) **(A)** or in low light (LL) (200 μmol photons m^–2^ s^–1^) **(B)** were exposed to light of various intensities for 3 h. All experiments were done using fully expanded leaves from the top of 4–5 weeks old plants. Before the measurement of Fv/Fm, leaves were dark-adapted for 30 min. Results represent the average and SD of three independent measurements. *, values are significantly different at *P* < 0.05 and **, values are significantly different at *P* < 0.01 between experimental groups.

Due to the light-induced production of a strong oxidant, P680^+^, photosystem II is the most susceptible electron transport complex to damage under HL stress (Huang et al., 2018). To determine the impact of varying light intensities on PSII stability, we exposed plants to various light intensities for 3 h and then determined their PSII efficiency after a dark adaptation period. As shown in Figure 2, both ML and LL grown plants having Chl *a/b* ratios of approximately 5 (CR L-I) had statistically significantly lower losses in PSII efficiency than WT or transgenic plants having Chl *a/b* ratios > 6.5, when exposed to light intensities ≥ 1,000 μmol photons m^–2^ s^–1^. Furthermore, plants having the smallest antenna size had the greatest PSII sensitivity to light stress.

### Changes in Various Factors Affecting NPQ

**FIGURE 3:**
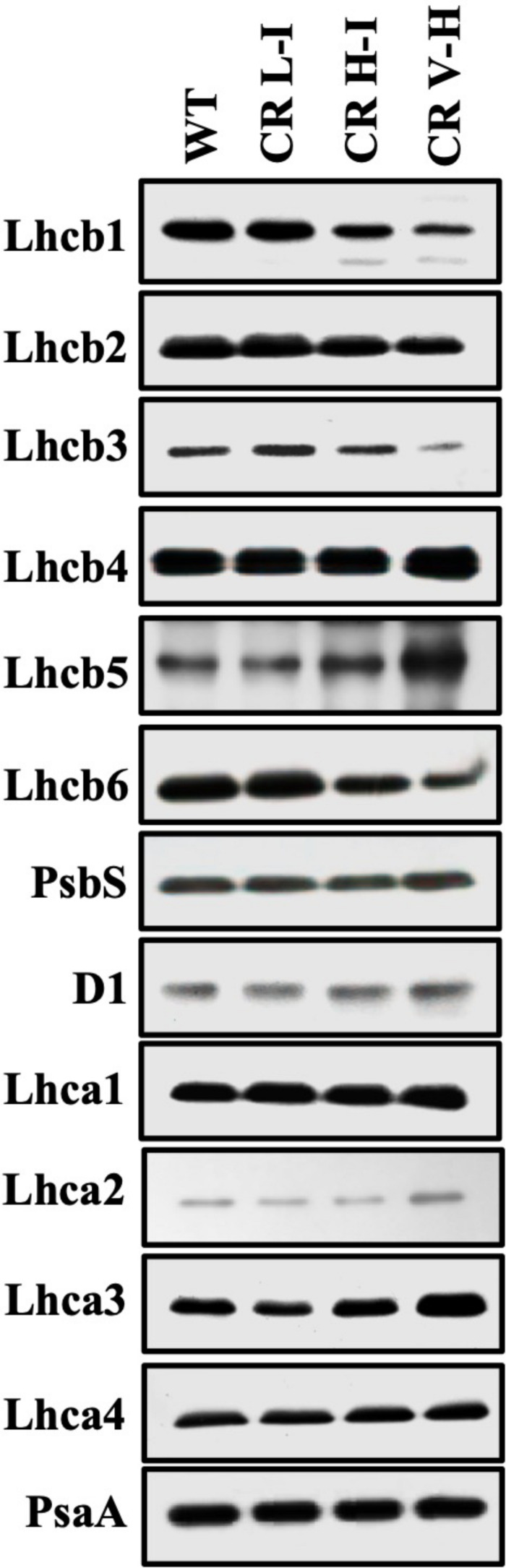
Comparison of thylakoid protein contents in wild-type and CAO RNAi transgenic lines. Thylakoid membranes were isolated from overnight dark-adapted wild-type (WT), CR L-I (Chl *a/b* = 4.5–6.5), CR H-I (Chl *a/b* = 6.5–8.5), and CR V-H (Chl *a/b* 8.5 or above) transgenic plant leaves. Thylakoid membrane contents 2 μg Chl were separated by the sodium dodecyl sulfate-polyacrylamide gel electrophoresis (SDS-PAGE) and immunoblotted using specific antibodies raised against Lhcb1, Lhcb2, Lhcb3, Lhcb4, Lhcb5, Lhcb6, Lhca1, Lhca2, Lhca3, Lhca4, D1, PsbS, and PsaA.

The LHCII is expected to be one of the major quenching site for NPQ (Horton et al., 1991). In our previous paper (Friedland et al., 2019), we observed that the reduction of LHCII trimer abundance was directly associated with reduced Chl *b* contents in CAO RNAi lines. To determine the impact of Chl b reduction on the specific levels of other LHC protein subunits we assessed the relative abundance of individual LHC proteins by immunoblotting (Figure 3). We observed a gradual reduction in major LHCII polypeptides (Lhcb1, Lhcb2, and Lhcb3) and especially Lhcb3 with reductions in Chl *b* levels. In contrast there was little impact on Lhcb4 and Lhcb5 and LHCI polypeptide (Lhca1, Lhca2, Lhca3, and Lhca4) levels as Chl *b* levels were reduced. The content of Lhcb6, a minor LHCII was also reduced in CR H-I and CR V-H lines, with no noticeable changes in the PSII and PSI core polypeptides, D1 and PsaA, respectively. Overall, it is apparent that Lhcb3 is most sensitive to reductions in Chl *b*. This is not surprising since it is the least abundant subunit in the LHCII complex. Given that Lhcb3 apparently evolved as plants adapted to land environments and is not found in algae, a role for this protein in NPQ mechanisms has been implied (Alboresi et al., 2008). Furthermore, the Lhcb3 protein is part of the M-trimer of LHCII family members (Crepin and Caffarri, 2018). Reductions in Lhcb3 content have been shown to be correlated with the loss of moderately bound LHCII M-trimers and alterations in the orientation of the M-trimers. Thus, Lhcb3 plays a critical role in regulating energy transfer from the LHC to the reaction center core in higher plants. While the greatest losses in LHCII protein subunit family member abundance were observed for Lhcb3 in low Chl *b* CAO RNAi lines, we also observed reductions in the abundance of Lhcb1 associated with reductions in Chl *b* content. Lhcb1 is predominantly found in the loosely bound LHCII L-trimers as well as in LHCII M-trimers. The reduction in Lhcb1 levels are consistent with previous descriptions demonstrating the loss of loosely bound LHCII trimers associated with the loss of Chl *b* (Friedland et al., 2019) as well as losses in Lhcb6 content involved in binding the LHCII M-trimer with PCII core. Overall, these results indicate that loosely bound LHCII L-trimers are most impacted by reductions in Chl *b* content followed by the LHCII M-trimers.

To determine the impact of Chl *b* reduction on NPQ levels, we measured PsbS and xanthophyll cycle carotenoid levels in the various CAO RNAi lines. The PsbS protein has been implicated in the regulation of NPQ (Li et al., 2002) and the xanthophyll cycle carotenoids participate in energy quenching (Niyogi et al., 1998). Significantly, there were no noticeable differences in the level of PsbS protein among the different CAO RNAi lines (Figure 3). This is surprising given the differential responses in NPQ levels among the different CAO RNAi lines to reductions in Chl *b* levels, thus implying that the observed perturbations in NPQ responses are not associated with changes in PsbS levels. Just as surprising, we observed that both zeaxanthin content per Chl *a* and the carotenoid de-epoxidation state increased in CAO RNAi lines with lower Chl *b* content (Table 1), indicating that steady state levels of zeaxanthin and the de-epoxidation state were also not correlated with NPQ (Figure 1). Overall, we observed about a 10 to 20% drop in the total xanthophyll cycle pigment content (violaxanthin + antheraxanthin + zeaxanthin) in all the CAO RNAi lines compared to WT plants. As antenna size became smaller neoxanthin and lutein content per Chl *a* also decreased, while the relative content of β-carotene per Chl *a* increased.

**TABLE 1:** Comparison of pigment compositions in wild-type and CAO RNAi transgenic lines under darkness and high light stress.

	**Neo**	**Vio**	**Ant**	**Lut**	**Zea**	**Chl *b***	**β-Car**	**Vio + Ant + Zea**	**AZ/VAZ**
WT dark	2.92 ± 0.78	10.95 ± 0.28	**ND**	20.12 ± 0.69	**ND**	27.67 ± 0.27	12.98 ± 0.30	10.95 ± 0.28	0
WT HL 1 h	2.79 ± 0.35	4.02 ± 0.01	2.19 ± 0.03	19.34 ± 0.33	4.07 ± 0.08	27.62 ± 0.16	12.43 ± 0.37	10.29 ± 0.05	0.50 ± 0.004
WT HL 2 h	3.34 ± 0.45	3.60 ± 0.01	2.43 ± 0.03	19.94 ± 0.56	5.40 ± 0.12	27.52 ± 0.36	13.06 ± 0.34	11.44 ± 0.10	0.58 ± 0.005
CR L-I dark	2.23 ± 0.25	9.32 ± 0.16	**ND**	18.04 ± 0.75	**ND**	16.83 ± 0.14	13.96 ± 0.22	9.32 ± 0.16	0
CR L-I HL 1 h	1.76 ± 0.23	2.62 ± 0.22	1.56 ± 0.07	17.23 ± 0.96	4.39 ± 0.27	16.45 ± 0.15	13.90 ± 0.33	8.57 ± 0.12	0.60 ± 0.028
CR L-I HL 2 h	2.00 ± 0.18	2.56 ± 0.30	1.45 ± 0.09	18.18 ± 0.71	5.15 ± 0.24	16.76 ± 0.31	14.55 ± 0.40	9.16 ± 0.17	0.64 ± 0.031
CR H-I dark	1.75 ± 0.09	8.57 ± 0.15	**ND**	16.69 ± 0.48	**ND**	13.34 ± 0.09	14.95 ± 0.25	8.57 ± 0.15	0
CR H-I HL 1 h	1.37 ± 0.26	2.23 ± 0.18	1.38 ± 0.07	15.90 ± 0.72	4.92 ± 0.18	13.29 ± 0.15	14.71 ± 0.41	8.53 ± 0.31	0.66 ± 0.010
CR H-I HL 2 h	1.35 ± 0.08	1.99 ± 0.10	1.35 ± 0.05	16.93 ± 0.67	5.98 ± 0.34	13.57 ± 0.19	15.33 ± 0.34	9.32 ± 0.25	0.71 ± 0.017
CR V-H dark	0.85 ± 0.11	9.91 ± 0.17	**ND**	14.07 ± 0.38	**ND**	6.91 ± 0.07	15.70 ± 0.68	9.91 ± 0.17	0
CR V-H HL 1 h	0.96 ± 0.12	2.08 ± 0.03	1.07 ± 0.17	15.82 ± 0.60	6.76 ± 0.35	7.29 ± 0.11	15.55 ± 0.87	9.91 ± 0.51	0.74 ± 0.007
CR V-H HL 2 h	0.94 ± 0.10	1.87 ± 0.14	0.72 ± 0.20	15.70 ± 0.35	7.12 ± 0.26	7.50 ± 0.09	15.82 ± 0.37	9.72 ± 0.19	0.77 ± 0.015

*Wild-type (WT), CR L-I (Chl *a/b* = 4.5–6.5), CR H-I (Chl *a/b* = 6.5–8.5) and CR V-H (Chl *a/b* 8.5 or above) plants were used, and overnight dark-adapted (dark) leaves from 4-week old Camelina plants were treated with high light (HL 1,000 μmol photons m^–2^ s^–1^) for 1 or 2 h. Pigments were subjected to high performance liquid chromatography analysis after extraction with 100% cooled acetone. Pigment content was normalized to 100 chlorophyll (Chl) *a* molecules. Ant, antheraxanthin; β-car, β-carotene; Lut, lutein; ND, not detectable; Neo, neoxanthin; Vio, violaxanthin; Zea, zeaxanthin. AZ/VAZ, de-epoxidation state was calculated from (0.5 Ant + Zea)/(Vio + Ant + Zea).*

### Relative Accumulation of Reactive Oxygen Species During Growth at Low and High Light Intensities as a Function of Antenna Size

**FIGURE 4:**
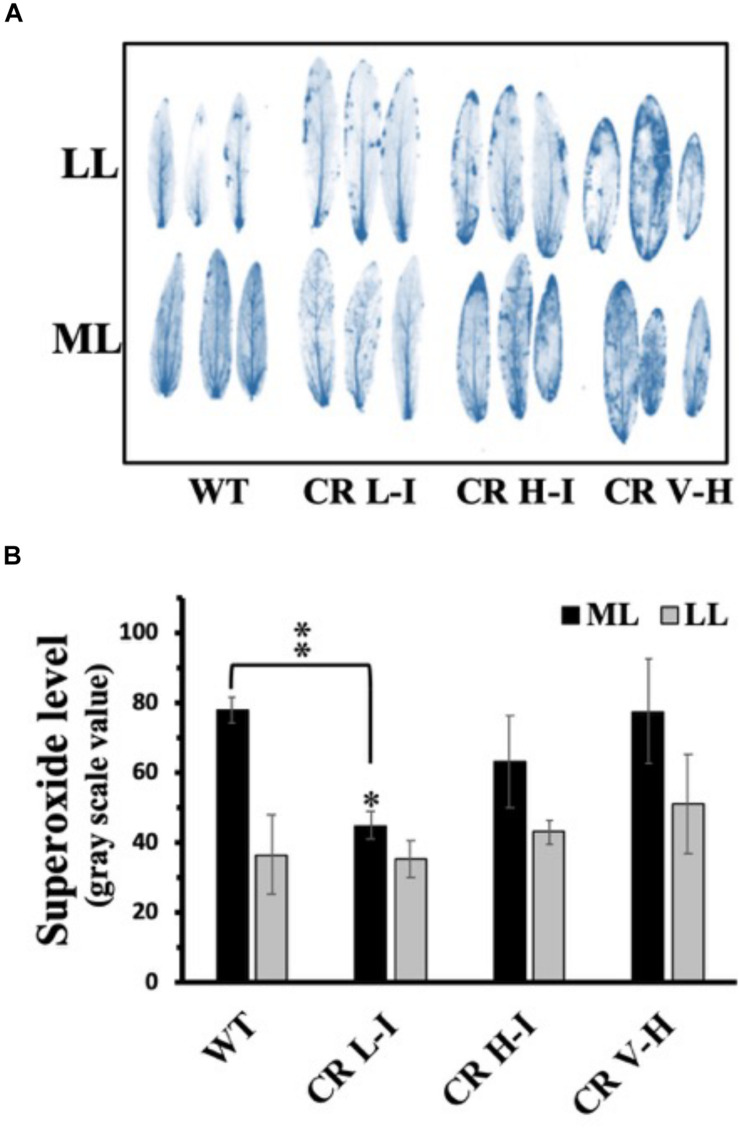
Comparison of superoxide anion radical production in wild-type and CAO RNAi transgenic lines. Moderate light (ML) (850 μmol photons m^–2^ s^–1^) and low light (LL) (200 μmol photons m^–2^ s^–1^) grown wild-type (WT), CR L-I (Chl *a/b* = 4.5–6.5), CR H-I (Chl *a/b* = 6.5–8.5) and CR V-H (Chl *a/b* 8.5 or above) transgenic plant leaves were treated with nitroblue tetrazolium (NBT) for histochemical staining for superoxide production during illumination at ML or LL for 4 h **(A)**. Quantitative levels of the superoxide produced were analyzed from the image in grayscale value using Image J (rsb.info.nih.gov/ij) **(B)**. All experiments were done using fully expanded leaves from the top of 4–5 weeks old plants. Results represent the average and SD of three independent measurements. *, values are significantly different at *P* < 0.05 and **, values are significantly different at *P* < 0.01 between experimental groups.

To assess the relative production of ROS during growth as a function of antenna size and light stress, we exposed plants grown at ML and LL to the corresponding light for 4 h followed by analysis of ROS accumulation (Asada, 1999; Bondarava et al., 2010; Zulfugarov et al., 2014). Fully expanded leaves were detached from overnight dark-adapted plants and chemical-infiltrated with ROS detection agents by floating on a 6 mM NBT solution in darkness for 2 h. We observed that NBT-detectable superoxide levels were lowest in the CR L-I line and greatest in WT and transgenic lines (CR H-I and CR V-H) having Chl *a/b* ratios > 6.5 following exposure of leaves from ML grown plants when to ML at 850 μmol photons m^–2^ s^–1^ for 4 h (Figure 4). In the rates of oxygen evolution with ferricyanide as a whole chain electron acceptor, we did not observe any significant differences among WT and all three transgenic lines (Supplementary Figure S2). In the case of the leaves from LL grown plants, exposed to LL at 200 μmol photons m^–2^ s^–1^, superoxide production in CR L-I lines was similar to that of WT but increased in CR H-I and CR V-H lines. As expected, plants grown under LL conditions had lower levels of ROS production than those grown under ML conditions suggesting ML grown plants are more predisposed to ROS production than LL grown plants. Thus, CAO RNAi lines having smaller antenna sizes were more prone to photodamage by ROS at both low and high light intensities.

### Lipid Peroxidation

**FIGURE 5:**
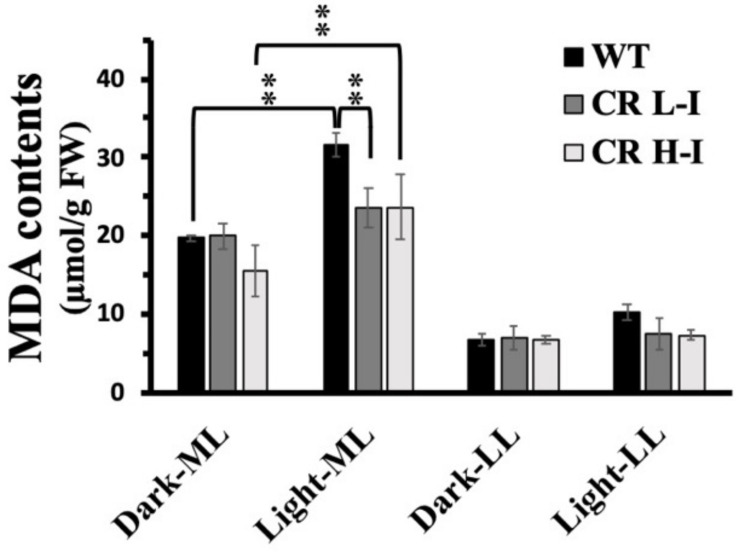
Comparison of malondialdehyde (MDA) content in wild-type and CAO RNAi transgenic lines. Moderate light (ML) (850 μmol photons m^–2^ s^–1^) and low light (LL) (200 μmol photons m^–2^ s^–1^) grown wild-type (WT), CR L-I (Chl *a/b* = 4.5–6.5), CR H-I (Chl *a/b* = 6.5–8.5), and CR V-H (Chl *a/b* 8.5 or above) transgenic plant leaves were exposed to growth light for 4 h. The MDA content was measured before the light illumination (Dark-ML or Dark-LL) and after the light illumination (Light-ML or Light-LL). All experiments were done using fully expanded leaves from the top of 4–5 weeks old plants. Results represent the average and SD of three independent measurements.**, values are significantly different at *P* < 0.05 between experimental groups.

One of the potential outcomes of high light stress induced ROS production is lipid peroxidation leading to the production of malondialdehyde (MDA) and damaged membranes. We measured lipid peroxidation levels in plants with different light harvesting antenna sizes as a function of light treatment by quantifying MDA content (Masia, 2003). As show in Figure 5, MDA levels in LL grown plants did not vary between WT, CR L-I and CR H-I lines when they were either dark-adapted or exposed to low light intensities for 4 h. In contrast, plants grown in medium light intensities and then exposed to the dark had twofold greater MDA levels then LL grown plants, suggesting that growth at higher light predisposes the plants to increased sustained MDA production. When dark-adapted, medium light grown plants were subsequently exposed to medium growth light for 4 h, MDA levels increased most substantially in WT plants (>60%) but to a lesser extent in the CR L-I and CR H-I lines (>20%). Increased MDA production in CR H-I plants was consistent with the elevated ROS production relative to the CR L-I transgenics as observed in Figure 4. These results indicate that WT plants exposed to greater than light saturating growth conditions generated substantially more ROS and MDA than the CR transgenics with smaller light harvesting antenna sizes. As expected, CAO RNAi plants having a range of light harvesting antenna sizes had lower MDA and ROS production levels when grown under sub-saturating light conditions (LL).

### Growth Light Intensity-Dependent Changes in the Seed Yield

**FIGURE 6:**
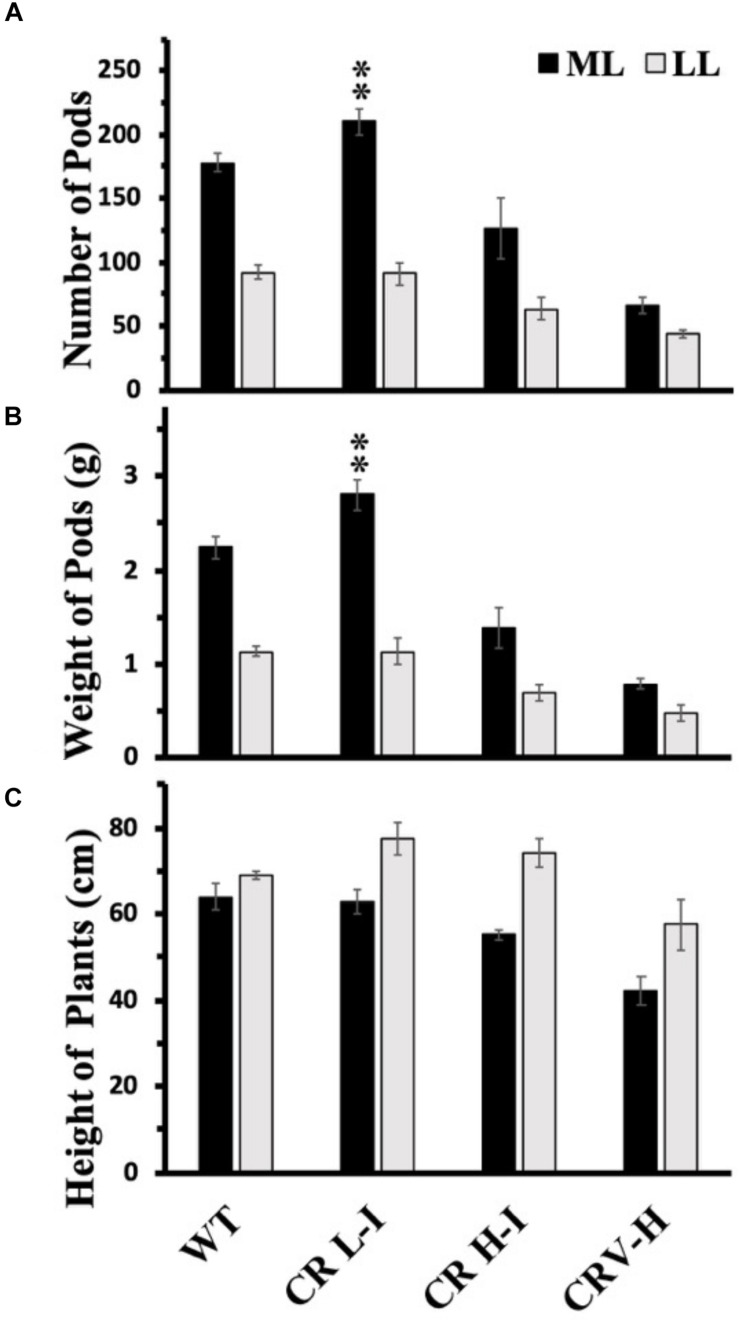
Comparison of number of seed pods **(A)**, weight of seed pods **(B)** and height of plants **(C)** in wild-type and CAO RNAi transgenic lines. Wild-type (WT), CR L-I (Chl *a/b* = 4.5–6.5), CR H-I (Chl *a/b* = 6.5–8.5), and CR V-H (Chl *a/b* 8.5 or above) transgenic lines grown in moderate light (ML) (850 μmol photons m^–2^ s^–1^) or in low light (LL) (200 μmol photons m^–2^ s^–1^). Grouping of transgenic lines was made using fully expanded leaves from the top of 4–5 weeks old plants. Results represent the average and SD of three independent measurements. **, values are significantly different at *P* < 0.05 between experimental groups.

Previously, we have demonstrated that Camelina plants having an upper canopy leaf Chl *a/b* ratio of 5 had the highest photosynthetic and biomass production yields (Friedland et al., 2019). To determine whether there was a relationship between light harvesting antenna size, growth light intensity and maximum seed yield, we compared the number of seed pods, total pod weight/plant and plant height between WT, CR L-I, CR H-I, and CR V-H plants grown either under ML or LL conditions (Figure 6). Previously, we had demonstrated that total harvestable seed yield in Camelina plants was largely determined by the number of seed pods and not seed mass or numbers of seeds per pod (Friedland et al., 2019). In the current study, the only observable statistically significant difference in yield was for number of pods and total pod yield/plant for the CR L-I plants versus WT when grown under ML growth conditions (Figure 6). Under ML conditions, both the number of seed pods and the total weight of pods were about 24% greater for the CR L-I line relative to WT. All plant lines grown at ML had greater pod numbers and yield than plants grown at LL but had identical plant height at both light intensities (Figure 6). Compared to WT, seed pod number, pod mass and plant height were all slightly reduced in CR H-I lines and further reduced in CR V-H lines when grown in LL conditions. These results demonstrate the impact of sink (pod) strength on determining the distribution of biomass allocation. Furthermore, it is apparent that WT light harvesting antenna sizes are most fit at low light intensities, and CAO RNAi plants having smaller light harvesting antenna sizes had reduced fitness compared to WT and the CR L-1 line.

### Relationship Between Chl *a/b* Ratios and Seed Yield

**FIGURE 7:**
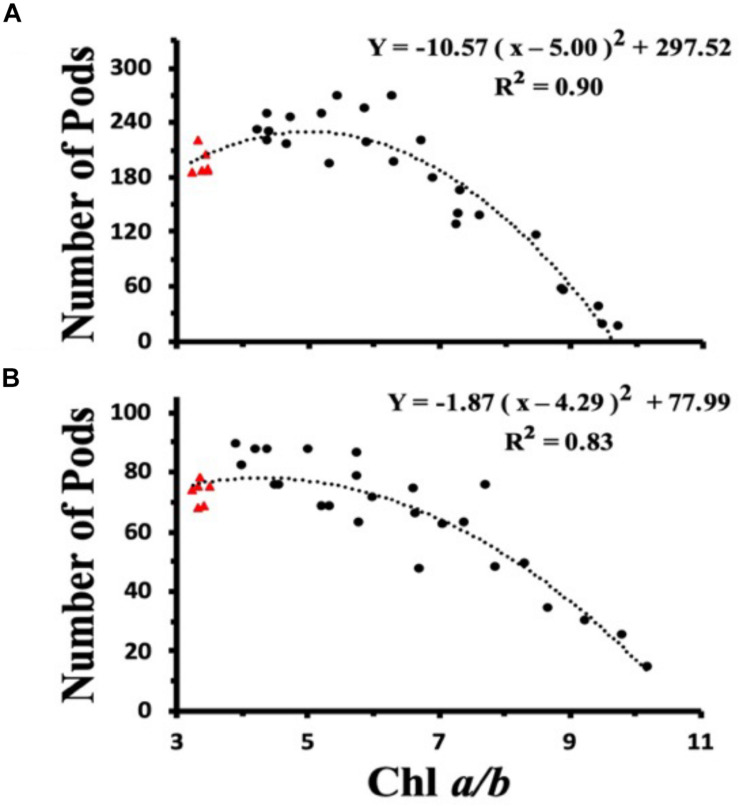
Chl *a/b* ratio dependent changes in seed pod number in wild-type (triangles) and CAO RNAi transgenic lines (circles) grown in moderate light (ML) (850 μmol photons m^–2^ s^–1^) **(A)** or in low light (LL) (200 μmol photons m^–2^ s^–1^) **(B)**. The data were fit with a second order polynomial function. Chl content was determined according to Porra et al. (1989).

To investigate the dependency of plant light harvesting antenna size on seed yield as a function of growth light intensities, we measured the number of seed pods/plant for WT and CAO RNAi plants across a range of Chl *a/b* ratios (measured at top fully expanded leaf) for ML and LL grown plants (Figures 7A,B). When the ML grown data were fit with a second order polynomial function, the peak seed pod yield was correlated with plants having a Chl *a/b* ratio of 5.0 (Figure 7A). However, when plants were grown under LL conditions, the peak seed pod yield was correlated with plants having a Chl *a/b* ratio of 4.3, more similar to WT plants (Figure 7B). In addition to the shift in optimal Chl *a/b* ratio or antenna size for plants grown under LL vs. ML conditions, there was a broadening in the distribution of seed yield vs. Chl *a/b* ratio for LL grown plants. Thus, more wild-type like Chl *a/b* ratios were associated with enhanced seed yield under low and medium light conditions while plants having Chl *a/b* ratios less than 5 had reduced seed yield or fitness.

## Discussion

Previously, it has been demonstrated that optimizing light harvesting antenna size enhances light utilization efficiency leading up to a 40% increase in algal biomass yield relative to WT green algae (Mussgnug et al., 2007; Perrine et al., 2012). Similar yield enhancement results were observed for transgenic Camelina having slightly reduced light harvesting antenna sizes (Friedland et al., 2019). It was observed both in green algae and plants that there is a tipping point in light harvesting antenna size (Chl *a/b* ratio = 5) where even a small change in Chl *a/b* values can result in significant differences in thylakoid membrane architecture, sensitivity to photoinhibition, and optimal biomass and seed yield (Perrine et al., 2012; Friedland et al., 2019). Interestingly, the transition point or the optimal Chl *a/b* ratio for photosynthetic performance for both green algae (Perrine et al., 2012) and Camelina (Friedland et al., 2019) is similar and more pronounced when grown under high light conditions.

In this study, we compared the light stress performance of Camelina CAO RNAi plants grown under ML and LL conditions to determine; (1) whether plants with different Chl *a/b* ratios and grown under different light intensities had altered sensitivity to photoinhibition associated with ROS production and lipid peroxidation; and (2) whether the optimal light harvesting antenna size for seed yield depends on the growth light intensities. Our results suggest that the optimal Chl *a/b* ratio for photosynthetic performance increases from that of wild-type plants as the growth light intensity increases. We observed that high-light induced photoinhibition, ROS and MDA production were lowest in ML grown plants having Chl *a/b* ratios (5) optimal for biomass production. In contrast, under low light growth conditions there were no significant differences in sensitivity to photoinhibition, ROS or MDA production between plants having Chl *a/b* ratios ranging from 3.2 (WT) to 10. As previously reported (Bruch and Thayer, 1983), high lipid peroxidation results in reduction in membrane fluidity by lipid peroxidation. Thus, plants with increased ROS production are likely to have more damaged membranes as observed in higher light grown plants (Figure 5).

The reason for the higher sensitivity to HL stress in plants having antenna sizes corresponding to Chl *a/b* ratios less than 5 may in part be due to the role of Chl *b* in stabilizing antennae pigment-protein complexes that mediate NPQ processes. The cbs3 *Chlamydomonas* lines lacking all Chl *b* showed impaired photo-autotropic growth and failed to carry out normal photoprotective processes including; state transitions and NPQ relative to WT plants (Perrine et al., 2012). Re-arrangement of PSII-LHCII supercomplexes is observed in HL-acclimated *Arabidopsis* plants, which is accompanied by reductions in Lhcb3 and Lhcb6 levels (Kouřil et al., 2013). In this study, we also report noticeable decreases in major LHCII polypeptides, especially Lhcb3 and Lhcb1 as well as a minor LHCII polypeptide, Lhcb6 (Figure 3). Lhcb3 is a major component of LHCII M-trimer, and Lhcb6 is involved in connecting the M-trimer to PSII core to form higher-order PSII-LHCII supercomplexes together with Lhcb4 (Kouřil et al., 2012). Lhcb1 levels were also reduced with increasing reductions in Chl *b*. Based on previous studies assessing the relative abundance of LHCII supercomplexes and LHCII loosely bound trimers (Friedland et al., 2019) we conclude that the LHCII L-trimers are most sensitive to reductions in Chl *b* followed by the LHCII M-trimers (Friedland et al., 2019). There is no direct evidence to prove whether missing Chl *b* was replaced by Chl *a*, however, during pigment assembly with LHC apoproteins, but we speculate that the pigment composition of LHCII is likely to have changed, because the ratio of Chl *a/b* in isolated LHCII-L trimer complexes was significantly increased [from 1.56 (WT) to 2.2 and 4.5] with decreased Chl levels (38 and 54%) in CR H-I and CR V-H lines (Friedland et al., 2019).

To determine the impact of Chl *b* and antenna size reduction on high-light induced photoprotective systems, we assessed the impact of varied Chl *b* reduction on NPQ associated factors. NPQ is enhanced by the presence of PsbS and zeaxanthin as well as by low pH (Li et al., 2002). Thus, we quantified the impact of Chl *b* reduction on PsbS and xanthophyll cycle carotenoid levels. The level of zeaxanthin per Chl *a* and the de-epoxidation state increased in the CR H-I and CR V-H lines (Table 1) inconsistent with the reduction in NPQ levels (Figure 1) and increased production of ROS in the low Chl *b* CAO RNAi lines (Figure 4). However, total xanthophyll pigment contents were lower than WT in CAO RNAi lines (Table 1) consistent with reductions in NPQ and increased ROS production. In contrast, there was no apparent reduction in PsbS levels in Camelina plants having wild-type to very low levels of Chl *b* (Figure 3). These results are surprising given that adjustments in PsbS levels in response to light stress are correlated with NPQ levels (Ballottari et al., 2007; Albanese et al., 2016). However, in other studies altering antenna size in *Arabidopsis* Lhcb1 and Lhcb2 knock-down mutants, no reduction in PsbS levels were observed (Nicol et al., 2019). We also observed reductions in Lhcb1 levels in the low Chl *b* CAO RNAi lines. Thus, it can be inferred that alterations in the loosely bound LHCII complex levels have little or no impact on PsbS levels or NPQ. Regardless, ROS production increased with reductions in Chl *b* levels without changes in NPQ levels. Collectively, these results suggest that ROS production and NPQ are not directly correlated and that alternate de-excitation pathways for NPQ and ROS production exist in plants (Berman et al., 2015). Overall, the lowest levels of ROS production were observed in WT and the CR L-1 lines and increased in very low Chl *b* lines. Consistent with earlier observations these results indicate the optimal light harvesting antenna size is a Chl *a/b* ratio of 5 (Friedland et al., 2019).

An additional factor that may determine the optimal light harvesting antenna size is the ability to recover from photoinhibitory light treatment. Under prolonged HL stress (24 h) the CR V-H lines failed to show even partial restoration of HL-repressed NPQ compared with WT, CR L-I and CR H-I lines (Figure 1). In fact, the CR L-I lines exhibited the greatest ability to recover NPQ activity following high light stress followed by WT, CR H-I and CR V-H. For high light grown plants, plants having a Chl *a/b* ratio of 5 had the greatest ability to recover NPQ following light stress, and the lowest yields of ROS, MDA and photoinhibition. Earlier studies had also shown that algae and plants with self-adjusting antenna sizes or Chl *a/b* ratio of 5, had the highest biomass productivity and seed production, respectively (Friedland et al., 2019; Negi et al., 2020). In this study, we compared seed production (fitness) across a broad range of plants having altered Chl *a/b* ratios grown at low and high light intensities. The assessment of various photosynthetic performance characteristics indicates that plants with Chl *a/b* ratios > 5 have reductions in multiple photosynthetic parameters leading to reduced fitness (seed yield).

In summary, CAO RNAi lines with slightly reduced antenna (Chl *a/b* ratio = 5) compared to WT showed improved tolerance to HL stress and recovered well following HL stress when grown at greater than saturating light intensities. These more efficient plants also accumulated lower levels of damaging ROS and demonstrated less damage (reduced MDA, reduced photoinhibition and greater NPQ recovery) than WT plants or plants with smaller antenna. The effects of small reductions in antenna size on excess light mediated stress response were not as pronounced for plants grown under LL conditions. In addition, there was a more pronounced tipping point in seed production (fitness) associated with slight antenna size reduction under high light than low light growth conditions. The fact that wild-type plants and algae have less than optimal antenna sizes, suggests that the driver for antenna size was to perform well under low light conditions rather than high light conditions.

## Data Availability Statement

All datasets generated for this study are included in the article/Supplementary Material.

## Author Contributions

GW contributed to the design of light stress experiments, and measurements of leaf chlorophyll content, MDA, superoxide and biomass yield, and substantially to drafting the manuscript. LM carried out light stress and leaf chlorophyll fluorescence measurement. RS provided transgenic plant materials and contributed to the experimental design, financial support, writing and editing of the manuscript. C-HL contributed to the design of the experimental system, provided research oversight and financial support, and manuscript editing.

## Conflict of Interest

The authors declare that the research was conducted in the absence of any commercial or financial relationships that could be construed as a potential conflict of interest.

## Footnotes

1 rsb.info.nih.gov/ij

## References

[B1] Albanese P. Manfredi M. Meneghesso A. Marengo E. Saracco G. Barber J. (2016). Dynamic reorganization of photosystem II supercomplexes in response to variations in light intensities. *Biochim. Biophys. Acta* 1857 1651–1660. 10.1016/j.bbabio.2016.06.011 27378191

[B2] Alboresi A. Caffarri S. Nogue F. Bassi R. Morosinotto T. (2008). In silico and biochemical analysis of Physcomitrella patens photosynthetic antenna: identification of subunits which evolved upon land adaptation. *PLoS One* 3:e0002033. 10.1371/journal.pone.0002033 18446222 PMC2323573

[B3] Asada K. (1999). The water-water cycle in chloroplasts: scavenging of active oxygens and dissipation of excess photons. *Annu. Rev. Plant Biol.* 50 601–639. 10.1146/annurev.arplant.50.1.601 15012221

[B4] Ballottari M. Dall’Osto L. Morosinotto T. Bassi R. (2007). Contrasting behavior of higher plant photosystem I and II antenna systems during acclimation. *J. Biol. Chem.* 282 8947–8958. 10.1074/jbc.M606417200 17229724

[B5] Berman G. P. Nesterov A. I. López G. V. Sayre R. T. (2015). Superradiance transition and nonphotochemical quenching in photosynthetic complexes. *J. Phys. Chem. C* 119 22289–22296. 10.1021/acs.jpcc.5b04455

[B6] Bondarava N. Gross C. M. Mubarakshina M. Golecki J. R. Johnson G. N. Krieger-Liszkay A. (2010). Putative function of cytochrome b559 as a plastoquinol oxidase. *Physiol. Plant.* 138 463–473. 10.1111/j.1399-3054.2009.01312.x 19947963

[B7] Bruch R. C. Thayer W. S. (1983). Differential effect of lipid peroxidation on membrane fluidity as determined by electron spin resonance probes. *Biochim. Biophys. Acta* 733 216–222. 10.1016/0005-2736(83)90525-4 6309228

[B8] Chen M. Li Y. Birch D. Willows R. D. (2012). A cyanobacterium that contains chlorophyll f–a red-absorbing photopigment. *FEBS Lett.* 586 3249–3254. 10.1016/j.febslet.2012.06.045 22796191

[B9] Crepin A. Caffarri S. (2018). Functions and evolution of Lhcb isoforms composing LHCII, the major light harvesting complex of photosystem II of green eukaryotic organisms. *Curr. Protein Pept. Sci.* 19 699–713. 10.2174/1389203719666180222101534 29473498

[B10] Friedland N. Negi S. Wu G. Ma L. Flynn S. Kummsa T. (2019). Fine-tuning the photosynthetic light harvesting apparatus for improved photosynthetic efficiency and biomass yield. *Sci. Rep.* 9 1–12. 10.1038/s41598-019-49545-8 31506512 PMC6736957

[B11] Fryer M. J. Oxborough K. Mullineaux P. M. Baker N. R. (2002). Imaging of photo-oxidative stress responses in leaves. *J. Exp. Bot.* 53 1249–1254. 10.1093/jexbot/53.372.1249 11997373

[B12] Gilmore A. M. Yamamoto H. Y. (1991). Zeaxanthin formation and energy-dependent fluorescence quenching in pea chloroplasts under artificially mediated linear and cyclic electron transport. *Plant Physiol.* 96 635–643. 10.1104/pp.96.2.635 16668233 PMC1080818

[B13] Hodges D. M. Delong J. M. Forney C. F. Prange R. K. (1999). Improving the thiobarbituric acid-reactive-substances assay for estimating lipid peroxidation in plant tissues containing anthocyanin and other interfering compounds. *Planta* 207 604–611. 10.1007/s004250050524 28456836

[B14] Hoober J. K. Eggink L. L. Chen M. (2007). Chlorophylls, ligands and assembly of light-harvesting complexes in chloroplasts. *Photosynth. Res.* 94 387–400. 10.1007/s11120-007-9181-1 17505910 PMC2117338

[B15] Horton P. Ruban A. V. Rees D. Pascal A. A. Noctor G. Young A. J. (1991). Control of the light-harvesting function of chloroplast membranes by aggregation of the LHCII chlorophyll—protein complex. *FEBS Lett.* 292 1–4. 10.1016/0014-5793(91)80819-o 1959588

[B16] Huang W. Zhang S.-B. Liu T. (2018). Moderate photoinhibition of photosystem II significantly affects linear electron flow in the shade-demanding plant Panax notoginseng. *Front. Plant Sci.* 9:637. 10.3389/fpls.2018.00637 29868090 PMC5962726

[B17] Kouřil R. Dekker J. P. Boekema E. J. (2012). Supramolecular organization of photosystem II in green plants. *Biochim. Biophys. Acta* 1817 2–12. 10.1016/j.bbabio.2011.05.024 21723248

[B18] Kouřil R. Wientjes E. Bultema J. B. Croce R. Boekema E. J. (2013). High-light vs. low-light: effect of light acclimation on photosystem II composition and organization in *Arabidopsis thaliana*. *Biochim. Biophys. Acta* 1827 411–419. 10.1016/j.bbabio.2012.12.003 23274453

[B19] Li X. P. Müller-Moulé P. Gilmore A. M. Niyogi K. K. (2002). PsbS-dependent enhancement of feedback de-excitation protects photosystem II from photoinhibition. *Proc. Natl. Acad. Sci. U.S.A.* 99 15222–15227. 10.1073/pnas.232447699 12417767 PMC137571

[B20] Masia A. (2003). “Physiological effects of oxidative stress in relation to ethylene in postharvest produce,” in *Postharvest Oxidative Stress in Horticultural Crops*, ed. Hodges D. M. (New York, NY: Food Products Press), 165–197.

[B21] Mussgnug J. H. Thomas-Hall S. Rupprecht J. Foo A. Klassen V. Mcdowall A. (2007). Engineering photosynthetic light capture: impacts on improved solar energy to biomass conversion. *Plant Biotechnol. J.* 5 802–814. 10.1111/j.1467-7652.2007.00285.x 17764518

[B22] Negi S. Perrine Z. Friedland N. Kumar A. Tokutsu R. Minagawa J. (2020). Light-regulation of light harvesting antenna size substantially enhances photosynthetic efficiency and biomass yield in green algae. *Plant J.* 10.1111/TPJ.14751 32180283

[B23] Nicol L. Nawrocki W. J. Croce R. (2019). Disentangling the sites of non-photochemical quenching in vascular plants. *Nat. Plants* 5 1177–1183. 10.1038/s41477-019-0526-5 31659240 PMC6861128

[B24] Niyogi K. K. Grossman A. R. Björkman O. (1998). Arabidopsis mutants define a central role for the xanthophyll cycle in the regulation of photosynthetic energy conversion. *Plant Cell* 10 1121–1134. 10.1105/tpc.10.7.1121 9668132 PMC144052

[B25] Ort D. R. Zhu X. Melis A. (2011). Optimizing antenna size to maximize photosynthetic efficiency. *Plant Physiol.* 155 79–85. 10.1104/pp.110.165886 21078863 PMC3014228

[B26] Peever T. L. Higgins V. J. (1989). Electrolyte leakage, lipoxygenase, and lipid peroxidation induced in tomato leaf tissue by specific and nonspecific elicitors from Cladosporium fulvum. *Plant Physiol.* 90 867–875. 10.1104/pp.90.3.867 16666890 PMC1061813

[B27] Perrine Z. Negi S. Sayre R. T. (2012). Optimization of photosynthetic light energy utilization by microalgae. *Algal Res.* 1 134–142. 10.1016/j.algal.2012.07.002 30363850

[B28] Porra R. Thompson W. Kriedemann P. (1989). Determination of accurate extinction coefficients and simultaneous equations for assaying chlorophylls a and b extracted with four different solvents: verification of the concentration of chlorophyll standards by atomic absorption spectroscopy. *Biochim. Biophys. Acta* 975 384–394. 10.1016/s0005-2728(89)80347-0

[B29] Towbin H. Staehelin T. Gordon J. (1979). Electrophoretic transfer of proteins from polyacrylamide gels to nitrocellulose sheets: procedure and some applications. *Proc. Natl. Acad. Sci. U.S.A.* 76 4350–4354. 10.1073/pnas.76.9.4350 388439 PMC411572

[B30] Zulfugarov I. S. Tovuu A. Eu Y.-J. Dogsom B. Poudyal R. S. Nath K. (2014). Production of superoxide from Photosystem II in a rice (*Oryza sativa* L.) mutant lacking PsbS. *BMC Plant Biol.* 14:242. 10.1186/s12870-014-0242-2 25342550 PMC4219129

[B31] Zulfugarov I. S. Tovuu A. Kim J.-H. Lee C.-H. (2011). Detection of reactive oxygen species in higher plants. *J. Plant Biol.* 54 351–357. 10.1007/s12374-011-9177-4

