# Supplementary material for: Identification of the Optimal Light Harvesting Antenna Size for High-Light Stress Mitigation in Plants
Source: Front Plant Sci. 2020 May 15;11:505. doi: 10.3389/fpls.2020.00505 (PMC7243658; doi:10.3389/fpls.2020.00505)
Supplement: Supplementary file 1 [file Table_1.docx]

**Figure S1.** Comparison of Chl *a/b* ratio in wild-type and CAO RNAi transgenic lines during growth in moderate light (ML) (850 *μ*mol photons m^-2^ s^-1^) and in low light (LL) (200 *μ*mol photons m^-2^ s^-1^). Wild-type (WT), CR L-I (Chl *a/b* = 4.5 - 6.5), CR H-I (Chl *a/b* = 6.5 - 8.5) and CR V-H (Chl *a/b* 8.5 or above) plants were used. Chl *a/b* ratios were measured using fully expanded leaves from the top of 3 - 5 weeks old plants, and grouping of transgenic lines was made using 3 weeks old plants. Results represent the average and SD of three independent measurements.

**Figure S2.** Comparison of oxygen evolution activity in wild-type and CAO RNAi transgenic lines. Thylakoid membranes were isolated from overnight dark-adapted wild-type (WT), CR L-I (Chl *a/b* = 4.5 - 6.5), CR H-I (Chl *a/b* = 6.5 - 8.5) and CR V-H (Chl *a/b* 8.5 or above) transgenic plant leaves as described by Gilmore et al. (1998). Oxygen evolution activity was measured using thylakoid membranes contents 10 *μ*g Chl in 1 mL reaction buffer containing 0.1 M sucrose, 10 mM NaCl, 10 mM KCl, 5 mM MgCl_2_, 10 mM Tricine, 1 mM KH_2_PO_4_ and 0.2% bovine serum albumin (pH 8.0) with 1 mM FeCN as an electron acceptor by illumination of a white light (800 *μ*mol photons m^−2^ s^−1^) for 5 min. Results represent the average and SD of three independent measurements.

**Reference**

Gilmore, A.M., Shinkarev, V.P., Hazlett, T.L., and Govindjee. (1998). Quantitative analysis of the effects of intrathylakoid pH and xanthophyll cycle pigments on chlorophyll a fluorescence lifetime distributions and intensity in thylakoids. *Biochemistry* 37, 13582-13593. doi: 10.1021/bi981384x
